# MiR-19b suppresses PTPRG to promote breast tumorigenesis

**DOI:** 10.18632/oncotarget.11799

**Published:** 2016-09-01

**Authors:** Minghui Liu, Rong Yang, Uzair Urrehman, Chao Ye, Xin Yan, Shufang Cui, Yeting Hong, Yuanyuan Gu, Yanqing Liu, Chihao Zhao, Liang Yan, Chen-Yu Zhang, Hongwei Liang, Xi Chen

**Affiliations:** ^1^ State Key Laboratory of Pharmaceutical Biotechnology, Jiangsu Engineering Research Center for MicroRNA Biology and Biotechnology, NJU Advanced Institute for Life Sciences (NAILS), School of Life Sciences, Nanjing University, Nanjing, Jiangsu 210046, China; ^2^ Department of Urology, The Affiliated Drum Tower Hospital of Nanjing University Medical School, Nanjing, Jiangsu 210008, China; ^3^ Department of Respiratory Medicine, The Affiliated Drum Tower Hospital of Nanjing University Medical School, Nanjing, Jiangsu 210008, China; ^4^ Provincial Key Laboratory of Biological Macro-molecules Research, Wannan Medical College, Wuhu, Anhui 241002, China

**Keywords:** miR-19b, PTPRG, proliferation, apoptosis, migration

## Abstract

Protein tyrosine phosphatase receptor type G (PTPRG) is an important tumor suppressor gene in multiple human cancers. In this study, we found that PTPRG protein levels were downregulated in breast cancer tissues while the mRNA levels varied irregularly, implying a post-transcriptional mechanism was involved. Because microRNAs are powerful post-transcriptional regulators of gene expression, we used bioinformatics analysis to search for microRNAs that potentially targets PTPRG in the setting of breast cancer. We identified two specific binding sites for miR-19b in the 3′-untranslated region of PTPRG. We further identified an inverse correlation between miR-19b and PTPRG protein levels, but not mRNA levels, in human breast cancer tissues. By overexpressing or knocking down miR-19b in MCF-7 cells and MDA-231 cells, we experimentally confirmed that miR-19b directly suppresses PTPRG expression. Furthermore, we determined that the inhibition of PTPRG by miR-19b leads to increased proliferation, stimulated cell migration and reduced apoptosis. Taken together, our findings provide the first evidence that miR-19b inhibits PTPRG expression to promote tumorigenesis in human breast cancer.

## INTRODUCTION

Breast cancer is the most commonly diagnosed invasive cancer worldwide and the second leading cause of cancer death among women. According to the cancer statistics of 2016, breast cancer alone is expected to account for 29% all new cancer diagnoses in women [1]. Therefore, it is a great urgency to clarify the mechanisms underlying the rapidly increasing incidence of breast cancer.

Protein tyrosine phosphorylation plays a crucial role in regulating biological processes directly relevant to cancer, such as proliferation, differentiation, apoptosis, migration and invasion. This dynamic process is governed by the balanced action of protein tyrosine kinases (PTKs) and protein tyrosine phosphatases (PTPs). PTKs catalyze the phosphorylation of tyrosine residues inside cells, whereas PTPs neutralize the effects of PTKs by selectively dephosphorylating their substrates [2]. Therefore, alterations in PTPs activity may affect cell growth, neoplastic processes and transformation.

Protein tyrosine phosphatase, receptor type G (PTPRG), also known as PTPγ, is a member of the PTP family. PTPRG has been identified as an important tumor suppressor gene. In recent decades, reduced expression of PTPRG has been frequently reported in multiple cancers, such as lung cancer, ovarian cancer and breast cancer [3–5]. However, the mechanism contributing to PTPRG silencing in cancers is largely unknown.

MicroRNAs (miRNAs) are small, single-stranded non-coding RNAs that regulate gene expression by binding to the 3′-untranslated regions (3′-UTR) of mRNA, eventually resulting in either degradation of the transcript or its translational inhibition. Aberrant miRNA expression has been reported to participate in the occurrence and development of many cancers. The miR-17-92 cluster is well known to act as key oncogenes during tumorigenesis. The enforced expression of the miR-17-92 cluster promotes the proliferation and suppresses the apoptosis of cancer cells and induces tumor angiogenesis [6, 7]. MiR-19b is an important component of the miR-17–92 cluster. The increased expression of miR-19b can promote tumorigenesis by repressing PTEN expression and activating the PI3K pathway [8, 9].

Although decreased PTPRG expression and increased miR-19b expression play important regulatory roles in carcinogenesis, little is known about the correlation between miR-19b and PTPRG in breast cancer. In this study, we predicted that PTPRG is a potential target gene of miR- 19b by bioinformatic analysis. Furthermore, we detected an inverse correlation between miR-19b and PTPRG expression in breast cancer tissues. Next, we experimentally confirmed the negative regulation of PTPRG by miR-19b in breast cancer cells. Finally, the biological roles of the miR-19b-mediated inhibition of PTPRG expression in human breast cancer were investigated.

## RESULTS

### Downregulation of PTPRG protein in human breast cancer tissues

**Figure 1:**
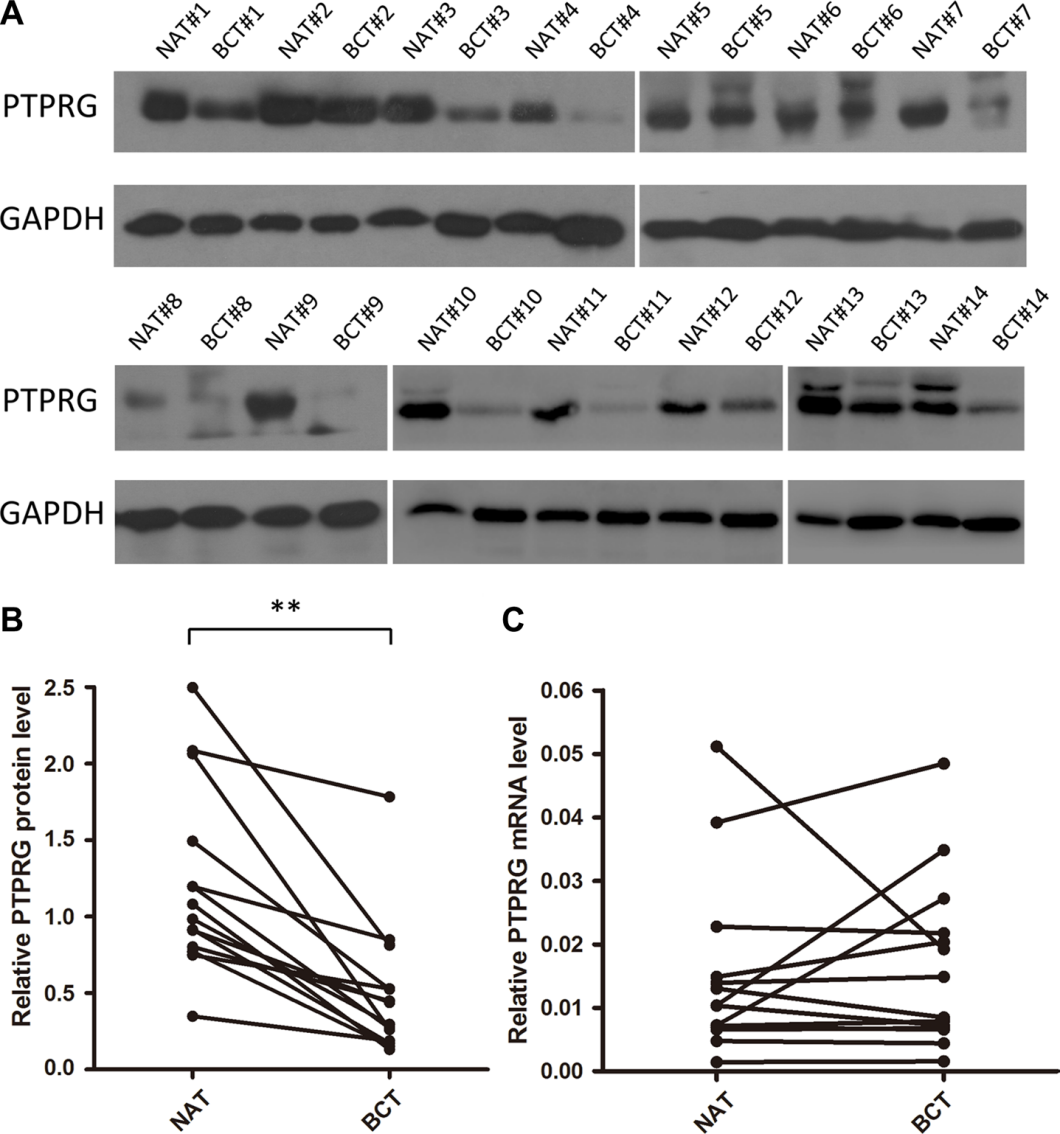
Western blotting analysis and quantitative analysis of PTPRG protein and mRNA levels in human breast cancer tissues (**A** and **B**) Western blotting analysis of PTPRG protein levels in 14 pairs of breast cancer tissues (BCT) and paired normal adjacent tissues (NAT). (**C**) Quantitative real-time PCR analysis of the relative PTPRG mRNA levels in the same 14 pairs of BCT and NAT tissue samples. **P < 0.01.

First, we determined the expression patterns of PTPRG in human breast cancer tissues. After measuring the protein levels of PTPRG in 14 paired breast cancer tissues and their corresponding noncancerous tissues, we observed that the PTPRG protein levels were dramatically reduced in the breast cancer tissues compared with the noncancerous tissues (Figure 1A and 1B). However, the PTPRG mRNA levels did not significantly differ between the cancerous and the noncancerous tissues (Figure 1C). This disparity between the protein and the mRNA PTPRG expression in human breast cancer suggests that a post-transcriptional mechanism is involved in the regulation of PTPRG.

### Identification of conserved miR-19b target sites within the 3′-UTR of PTPRG

**Figure 2:**
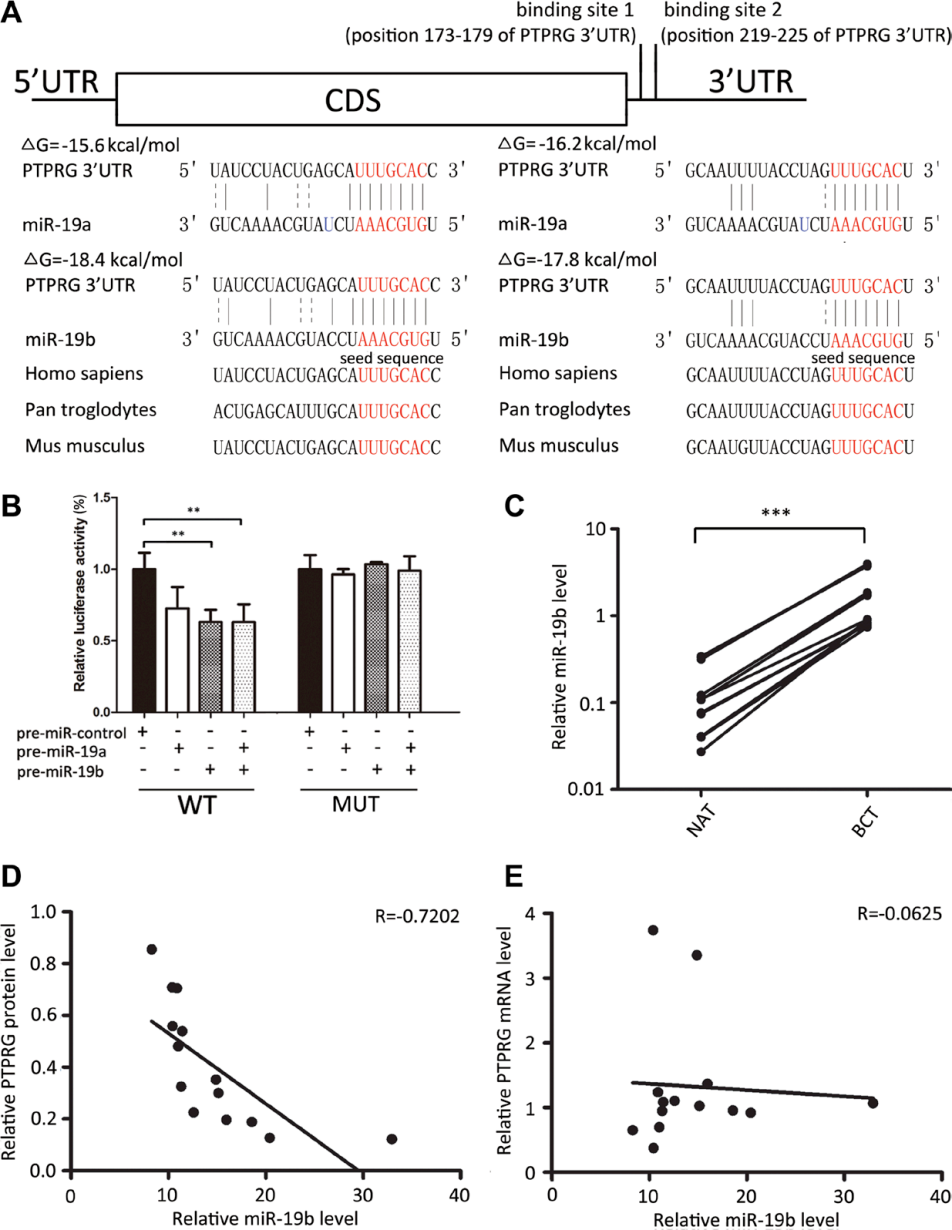
Inverse correlation between miR-19b and PTPRG protein levels in human breast cancer tissues (**A**) Schematic description of the hypothesized duplexes formed by the interaction between the binding sites of the PTPRG 3′-UTR (top) and miR-19a/b (bottom). The predicted free energy value of each hybrid was indicated. The seed-binding sites were demarked and all nucleotides in these regions were completely conserved in several species. (**B**) Direct recognition of the PTPRG 3′-UTR by miR-19a/b. MCF-7 cells were co-transfected with a firefly luciferase reporter containing either the wild-type (WT) or mutant (MUT) miR-19a/b binding sites in the PTPRG 3′-UTR and with either pre-miR-control or pre-miR-19a/b. The cells were assessed using a luciferase assay kit 24 h after transfection,. The results are displayed as the ratio of firefly luciferase activity in the miR-19a/b-transfected cells to that in the control cells. (**C**) Quantitative RT-PCR analysis of the miR-19b levels in 14 pairs of BCT and NAT tissue samples. (**D**) Pearson's correlation scatter plot of the fold change of miR-19b and PTPRG protein in human breast cancer tissues. (**E**) Pearson's correlation scatter plot of the fold change of miR-19b and PTPRG mRNA in human breast cancer tissues. ***P < 0.001; **P < 0.01.

MiRNAs repress the expression of mRNA transcripts at the post-transcriptional level. Thus, miRNAs may be a biologically relevant regulator of PTPRG expression in human breast cancer. To identify potential miRNAs that target PTPRG, three computational algorithms, including TargetScan [10], miRanda [11] and PicTar [12], were used in combination. Through these approaches, about 20 miRNAs were identified as potential PTPRG regulators. After removing the miRNAs previously reported to be tumor suppressor or increased in breast cancer, miR- 19a and miR-19b (miR-19a/b) were finally selected as candidate regulatory miRNAs of PTPRG. MiR-19a/b differs only in one nucleotide and they generally share common target mRNAs. The predicted interaction between miR-19a/b and the target sites within the PTPRG 3′-UTR is illustrated in Figure 2A. Two potential miR-19a/b targeting sites were found in the 3′-UTR of the PTPRG mRNA sequence and were close but non-overlapping. The minimum free energy values of the hybrids between miR- 19b and the binding sites on the PTPRG 3′-UTR are −18.4 and −17.8 kcal/mol and are lower than those of miR- 19a (−15.6 and −16.2 kcal/mol), suggesting that miR- 19b may bind more tightly to PTPRG 3′-UTR than miR-19a. Furthermore, the miR-19a/b binding sequences in the PTPRG 3′-UTR were highly conserved across species.

To determine whether miR-19a/b directly targeted PTPRG by binding to the presumed sites in the 3′-UTR of the PTPRG mRNA, the full-length PTPRG 3′-UTR containing the sole presumed miR-19a/b binding sites was fused downstream of the firefly luciferase gene in a reporter plasmid. The resulting plasmid was co-transfected into the MCF-7 cells along with both a transfection control plasmid (β-gal) and pre-miR-19a/b (or a scrambled negative control RNA). As expected, luciferase reporter activity was significantly reduced in the cells transfected with pre-miR-19a/b (Figure 2B). Interestingly, co-treatment with pre-miR-19b suppressed the luciferase reporter activity to a greater extent than with pre-miR-19a (Figure 2B) again suggesting that PTPRG is more likely to be a bona fide target of the miR-19b rather than miR-19a. Thus, we focused on miR-19b in further studies. Next, we introduced point mutations into the corresponding complementary sites in the 3′-UTR of PTPRG to eliminate the predicted miR-19b binding sites. This mutated luciferase reporter was unaffected by the overexpression of miR-19b (Figure 2B). This finding suggests that the putative binding sites of PTPRG strongly contribute to the interaction between miR-19b and PTPRG mRNA. Therefore, our results indicate that miR-19b directly inhibits PTPRG translation, resulting in the suppression of PTPRG expression.

### Detection of an inverse correlation between the miR-19b levels and the PTPRG protein levels in breast cancer tissues

MiRNAs are generally thought to have expression patterns that are the opposite of their targets. We next investigated whether miR-19b was inversely correlated with PTPRG in cancer tissues. After determining the levels of miR-19b in the same 14 pairs of breast cancer tissues and their corresponding noncancerous tissues, we showed that miR-19b levels were consistently increased in breast cancer tissues (Figure 2C). The inverse correlation between miR-19b and PTPRG protein levels (Figure 2D) and the disparity between the miR-19b and PTPRG mRNA levels (Figure 2E) were further illustrated using Pearson's correlation scatter plots. The data indicated that miR-19b mediates the post-transcriptional repression of PTPRG gene expression in human breast cancer. Thus, PTPRG was deduced to be a miR-19b target based on both computational predictions and the inverse correlation between the levels of miR-19b and PTPRG protein, but not mRNA levels, in human breast cancer.

### Validation of miR-19b as a negative regulator of PTPRG

**Figure 3:**
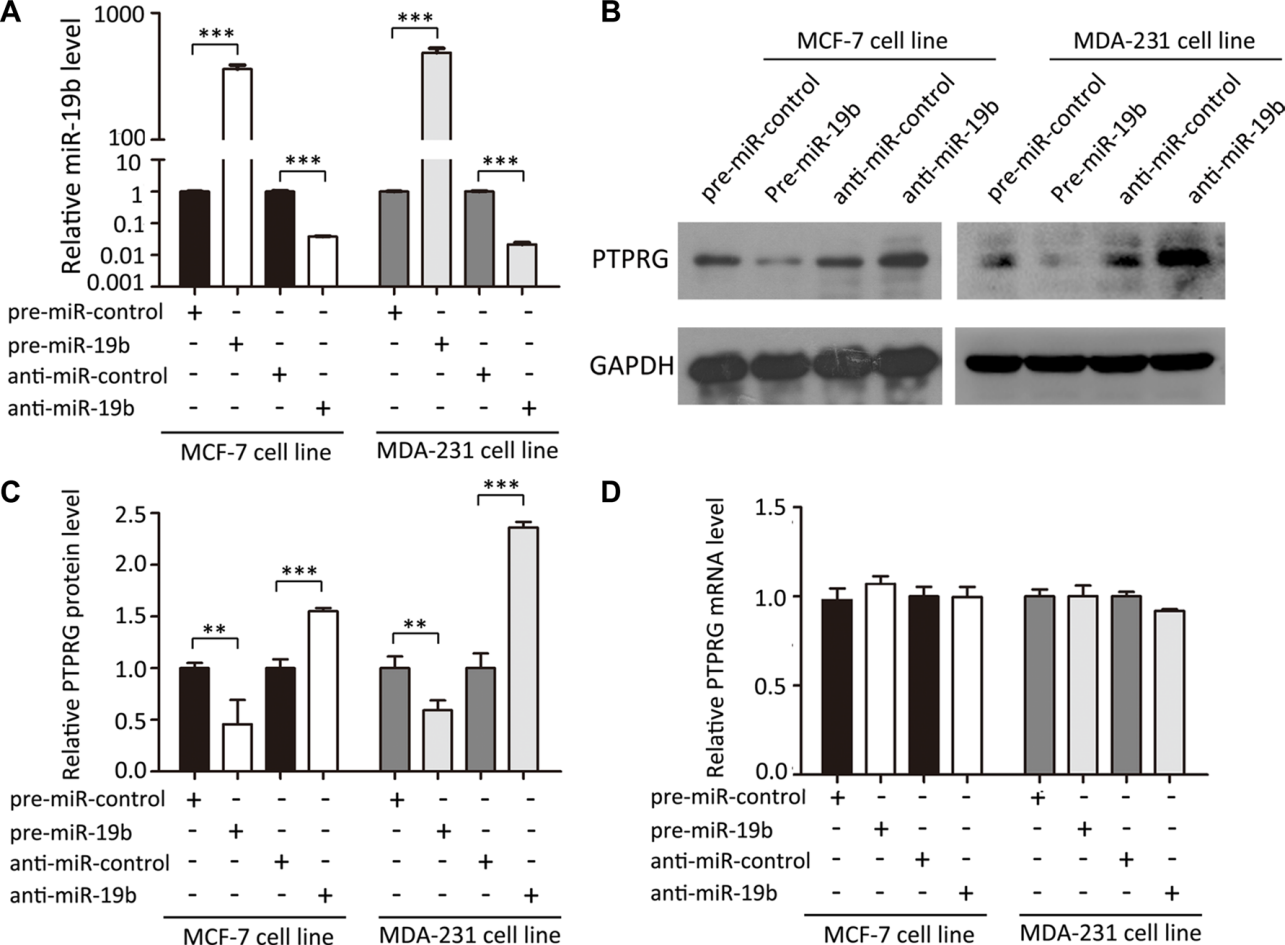
MiR-19b directly regulates PTPRG expression at the post-transcriptional level (**A**) Quantitative RT-PCR analysis of miR-19b levels in MCF-7 and MDA-231 cells treated with pre-miR-control, pre-miR-19b, anti-miR-control or anti-miR-19b. (**B** and **C**) Western blotting analysis of PTPRG protein levels in MCF-7 and MDA-231 cells treated with pre-miR-control, pre-miR-19b, anti-miR-control or anti-miR-19b. (**D**) Quantitative RT-PCR analysis of PTPRG mRNA levels in MCF-7 and MDA-231 cells treated with pre-miR-control, pre-miR-19b, anti-miR-control or anti-miR-19b. ***P < 0.001; **P < 0.01.

The correlation between miR-19b and PTRPG was further examined by assessing PTPRG expression in the human breast cancer cell line MCF-7 and MDA- 231 after overexpressing or knocking down miR-19b. In these experiments, miR-19b overexpression was achieved by transfecting MCF-7 and MDA-231 cells with pre-miR- 19b (a synthetic RNA oligonucleotide duplex mimicking the miR-19b precursor), whereas miR-19b knockdown was achieved by transfecting the cells with anti-miR- 19b (a chemically modified antisense oligonucleotide designed to specifically target mature miR-19b). The efficient overexpression or knockdown of miR-19b is shown in Figure 3A. Clearly, the miR-19b levels were significantly increased in the MCF-7 and MDA-231 cells when these cells were transfected with pre-miR-19b, whereas the miR-19b levels were decreased when the cells were transfected with anti-miR-19b. As anticipated, the PTPRG protein levels were significantly reduced by the overexpression of miR-19b and increased by the knockdown of miR- 19b (Figure 3B and 3C) in MCF- 7 and MDA-231 cells. To determine the level at which miR-19b affected PTPRG expression, we repeated the above experiments and examined the expression levels of PTPRG mRNA. The alteration of miR-19b levels did not influence the mRNA levels of PTPRG (Figure 3D). These results prove that miR-19b regulates PTPRG expression at the post-transcriptional level.

### MiR-19b promotes the proliferation and migration and inhibits the apoptosis of breast cancer cells by targeting PTPRG

Subsequently, we analyzed whether the knockdown of PTPRG affects proliferation, migration and apoptosis in MCF-7 and MDA-231 cells. SiRNA sequences targeting different sites of human PTPRG cDNA were designed to knock down PTPRG expression Supplementary Figure S1I. The efficient transfections are shown in Supplementary Figure S1A–S1C. Consistent with previous studies showing that PTPRG is a candidate tumor suppressor, MCF-7 and MDA-231 cells transfected with PTPRG siRNA showed significantly increased proliferation (Supplementary Figure S1D). Moreover, the cells transfected with PTPRG siRNA showed stimulated cell migration (Supplementary Figure S1E and S1G) and reduced apoptosis (Supplementary Figure S1F and S1H).

**Figure 4:**
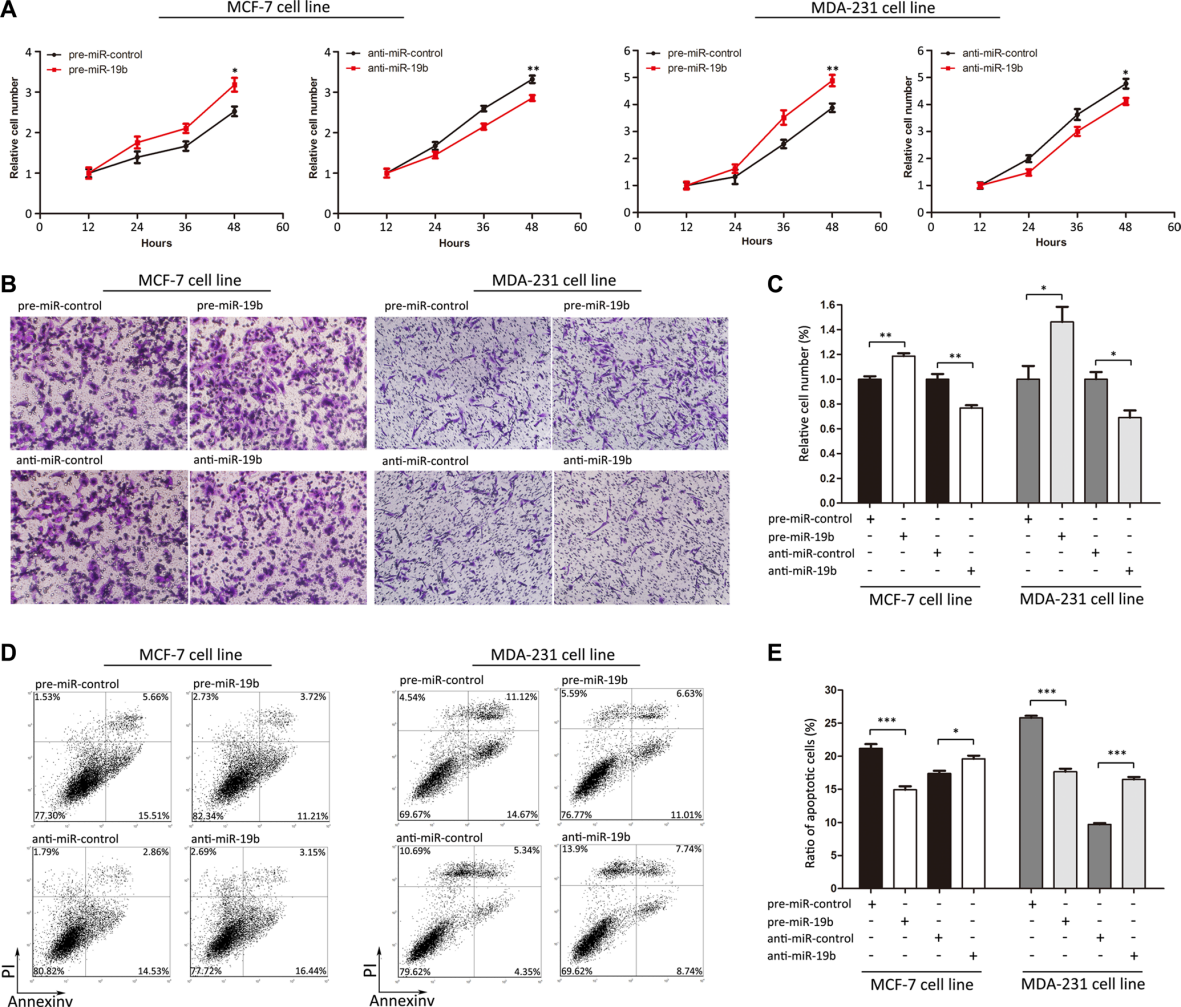
Effects of miR-19b on the proliferation, migration and apoptosis of MCF-7 and MDA-231 cells (**A**) Cell viability assays were performed 12, 24, 36 and 48 h after the transfection of MCF-7 and MDA-231 cells with pre-miR-control, pre- miR- 19b, anti-miR-control or anti-miR-19b. (**B** and **C**) Transwell migration assays were performed after the transfection of MCF-7 and MDA-231 cells with pre-miR-control, pre-miR-19b, anti-miR-control or anti-miR-19b. (**D** and **E**) Apoptosis assays were performed after the transfection of MCF-7 and MDA-231 cells with pre-miR-control, pre-miR-19b, anti-miR-control or anti-miR-19b. ***P < 0.001; **P < 0.01; *P < 0.05.

We next focused on the biological functions of miR-19b in regulating PTPRG in MCF-7 and MDA- 231 cells. The data showed that MCF-7 and MDA-231 cells transfected with pre-miR-19b exhibited increased proliferation, whereas transfecting with anti-miR-19b had the opposite effect on cell proliferation (Figure 4A). Moreover, the cells transfected with pre-miR-19b showed stimulated cell migration, whereas the cells transfected with anti-miR-19b exhibited inhibited migration (Figure 4B and 4C). Similarly, the cells transfected with pre-miR-19b showed reduced apoptosis, whereas the cells transfected with anti-miR-19b exhibited increased apoptosis (Figure 4D and 4E). These results suggested that miR-19b can promote proliferation and migration and inhibit the apoptosis of breast cancer cells.

## DISCUSSION

During the past two decades, the incidences of breast cancer continue to increase in both developing and developed countries. Besides, although the breast cancer mortality rates have declined significantly due to improved diagnostic and prognostic techniques, drug resistance and limited comprehension of breast cancer biology still limit the efficiency of treatments. Thus, it is of great clinical significance to explore the mechanism of breast carcinogenesis and to seek potential treatment for early intervention in breast cancer.

Aberrant tyrosine phosphorylation plays an important role in the tumorigenesis of human breast cancer. Recently, a member of the PTP family, PTPRG, has emerged as an estrogen-regulated tumor suppressor gene in human breast cancer [3]. Lower expression levels of PTPRG were found in breast cancer, which caused uncontrolled cell proliferation. However, the mechanism of PTPRG silencing in breast cancer is not clear. In this study, we found an alternative mechanism in which increased miR-19b negatively regulates PTPRG expression at the post-transcriptional level in human breast cancer.

Because PTPRG is an important tumor suppressor that is frequently deleted in multiple tumors, it may be a potential new target for breast cancer therapy. However, technical limitations make it difficult to stably express PTPRG *in vivo*. Considering that miR-19b is an upstream regulator of PTPRG, it is possible to downregulate miR-19b for restoration of PTPRG expression *in vivo*. Currently, some preclinical studies have shown the potential of miRNAs as a therapeutic target of human cancers. Further studies should be conducted to characterize the feasibility of targeting miR-19b in breast cancer therapy and develop simplified and cost-effective manipulation methods.

As a key oncogenic component of the miR-17- 92 cluster, overexpression of miR-19 is implicated in carcinogenesis processes. For example, miR-19b promotes the tumorigenicity of Myc-driven B-cell lymphomas by targeting PTEN [13]. MiR-19b also contributes to human cervical carcinoma cellular proliferation and invasion through suppressing vasopressin-activated calcium-mobilizing receptor-1 (CUL5) [14]. In breast cancer, upregulation of miR-19 suppresses downstream proteins, including PTEN, p-AKT, p-MDM2 and p53, leading to breast cancer carcinogenesis. Moreover, the breast-cancer-promoting role of miR-19/PTEN/AKT/p53 axis can be reversed by curcumin, implying that miR-19 is a potential target for breast cancer intervention [15]. In this study, we identified a novel relationship between miR-19b and PTPRG in human breast cancer. Because PTPRG is involved in reversible tyrosine phosphorylation, the influence of miR-19b on tumorigenesis and other biological processes may be momentous. In agreement with this, miR-19b was found herein to directly suppress PTPRG expression and consequently promote cell proliferation and migration and inhibits the apoptosis of breast cancer cells. Because PTPRG reduction can mimic miR-19b induction in promoting breast cancer cell proliferation and migration and suppressing apoptosis, it is possible that targeting PTPRG is one mechanism by which miR-19b exerts its oncogenic function. Therefore, modulation of PTPRG by miR-19b may explain, at least in part, why the upregulation of miR-19b can promote tumor growth and breast cancer formation.

Taken together, this study provides the first evidence that miR-19b reduces PTPRG expression at the post-transcriptional level to promote tumorigenesis in human breast cancer. This finding promotes our understanding of breast cancer biology and may provide advancements in the diagnosis and treatment for early intervention in breast cancer.

## MATERIALS AND METHODS

### Human tissue

Breast cancer tissues (BCT) and paired normal adjacent tissues (NAT) were acquired from patients undergoing a surgical procedure at the Affiliated Gulou Hospital of Nanjing University (Nanjing, China). Both tumor tissues and noncancerous tissues were histologically analyzed for diagnostic confirmation. The pathological type of each cancer was confirmed to be adenocarcinoma. Written consent was provided by all of the patients or their guardians, and the Ethics Committee of the Affiliated Gulou Hospital of Nanjing University approved all aspects of this study. The tissue fragments were immediately frozen in liquid nitrogen at the time of surgery and stored at −80°C. The clinical features of the patients are listed in Supplementary Table S1.

### Cell culture

The human breast cancer cell lines, MCF-7 and MDA-231, were purchased from the Shanghai Institute of Cell Biology, Chinese Academy of Sciences (Shanghai, China). The MCF-7 cells were cultured in DMEM medium (Gibco, Carlsbad, CA, USA) supplemented with 10% fetal bovine serum (Gibco) in a humidified incubator at 37°C with 5% CO_2_. The MDA-231 cells were cultured in L-15 medium (Gibco, Carlsbad, CA, USA) supplemented with 15% fetal bovine serum (Gibco) in a humidified incubator at 37°C with 5% CO_2_.

### RNA isolation and quantitative RT-PCR

Total RNA was extracted from the cultured cells and tissues using TRIzol Reagent (Invitrogen) according to the manufacturer's instructions. Assays to quantify mature miRNAs were performed using TaqMan miRNA probes (Applied Biosystems, Foster City, CA, USA) according to the manufacturer's instructions. Briefly, 1 μg of total RNA was reverse-transcribed to cDNA using AMV reverse transcriptase (TaKaRa, Dalian, China) and a stem-loop RT primer (Applied Biosystems). The reaction conditions were as follows: 16°C for 30 min, 42°C for 30 min, and 85°C for 5 min. Real-time PCR was performed using a TaqMan PCR kit on an Applied Biosystems 7500 Sequence Detection System (Applied Biosystems). The reactions were incubated in a 96-well optical plate at 95°C for 10 min, followed by 40 cycles of 95°C for 15 s and 60°C for 1 min. All of the reactions were executed in triplicate. The cycle threshold (C_T_) data were determined using fixed threshold settings after the reactions were complete, and the mean C_T_ was determined from triplicate PCRs. A comparative C_T_ method was used to compare each condition with the controls. U6 snRNA was used as an internal control, and the relative amount of miRNA normalized to the U6 snRNA levels was calculated using the equation 2^−ΔΔCT^, in which ΔΔC_T_= (C_T miRNA_ - C_T U6_) _target_ - (C_T miRNA_ - C_T U6_) _control_.

To quantify the PTPRG and GAPDH mRNA levels, 1 μg of total RNA was reverse-transcribed to cDNA using Oligo d(T)18 primers (TaKaRa) and ThermoScript reverse transcriptase (Invitrogen). The reaction conditions were as follows: 42°C for 60 min and 70°C for 10 min. Then, Real-time PCR was performed with the RT product, SYBR Green dye (Invitrogen) and specific primers for PTPRG or GAPDH. The sequences of the primers were as follows: PTPRG sense: 5′-TTGGGATCATAACGCACAGA-3′; PTPRG antisense: 5′-CTCGACTTGGCCAGTACACA-3′; and GAPDH sense: 5′-GATATTGTTGC CATCAATGAC-3′; GAPDH antisense: 5′-TTGATTTTGGAGGGATCTCG-3′. The reactions were incubated at 95°C for 5 min, followed by 40 cycles of 95°C for 30 s, 60°C for 30 s and 72°C for 1 min. After the reactions were completed, the C_T_ values were determined by setting a fixed threshold. The relative amount of PTPRG mRNA was normalized to the GAPDH mRNA level.

### MiRNA overexpression or knockdown

MiRNA overexpression was achieved by transfecting the cells with a miRNA mimic (a synthetic RNA oligonucleotide duplex mimicking the miRNA precursor sequence), whereas knockdown was achieved by transfecting the cells with a miRNA inhibitor (a chemically modified, single-stranded antisense oligonucleotide designed to specifically target a mature miRNA). Synthetic RNA molecules, including pre- miR- 19b and a scrambled negative control RNA (pre- miR-control and anti-miR- control), were purchased from GenePharma (Shanghai, China). The MCF-7 and MDA-231 cells were seeded on 6-well plates and transfected with Lipofectamine 2000 (Invitrogen) the next day when the cells were approximately 70% confluent. For the overexpression experiments of miRNA, 100 pmol of pre-miR-19b was used, and for the knockdown experiments of miRNA, 100 pmol of anti-miR-19b was used. After 6 h, the medium of the MCF-7 and MDA-231 cells was changed to DMEM or L-15, respectively, supplemented with 2% fetal bovine serum. The cells were harvested at 24 h and 48 h after transfection for the isolation of total RNA and protein, respectively.

### SiRNA interference assay

The siRNA sequences were as follows: siRNA sense: 5′-CCUUCUGAAAGACGACUAUTT-3′; and siRNA antisense: 5′-AUAGUCGUCUUUCAGAAGGTT-3′. A scrambled siRNA (Invitrogen) was used as a negative control. The overexpression plasmid or siRNA was transfected into the MCF-7 and MDA-231 cells using Lipofectamine 2000 (Invitrogen) according to the manufacturer's instructions. Total RNA and protein were isolated at 24 h and 48 h after transfection. The PTPRG mRNA and protein expression levels were assessed by quantitative RT-PCR and Western blotting, respectively.

### Luciferase reporter assay

The entire 3′-UTR of human PTPRG was amplified by PCR using human genomic DNA as a template. The PCR products were inserted into the p-MIR-reporter plasmid (Ambion, Austin, TX, USA). The insertion was confirmed to be successful by DNA sequencing. To evaluate the binding specificity, the sequences that interact with the seed sequence of miR-19b were mutated (from TTTGCAC to AAACGTG), and the mutant PTPRG 3′- UTR was inserted into an equivalent luciferase reporter plasmid. For the luciferase reporter assay, the cells were seeded into 6-well plates and co-transfected with 2 μg of a firefly luciferase reporter plasmid, 2 μg of a β-galactosidase (β-gal) expression plasmid (Ambion), and equal amounts (100 pmol) of miR-19b mimic, inhibitor, or a scrambled negative control RNA using Lipofectamine 2000 (Invitrogen). The β-gal plasmid was used as a transfection efficiency control. The cells were harvested 24 h after transfection and were analyzed for luciferase activity using luciferase assay kits (Promega, Madison, WI, USA).

### Protein isolation and Western blotting

Cells or tissues were lysed in RIPA lysis buffer (50 mM Tris-HCl, 150 mM NaCl, 0.1% SDS, 1% NP-40, 0.25% Sodium deoxycholate and 1 mM EDTA, pH 8.0) with freshly added protease inhibitor cocktail (Roche) for 30 min on ice, and then centrifuged at 16,000 × g at 4°C for 10 min. The supernatant was collected, and the protein concentration was calculated with a BCA protein assay kit (Thermo Scientific, Rockford, IL, USA). The proteins were separated via SDS-PAGE (Bio-Rad). After electrophoresis, the proteins were electrotransferred to PVDF membranes (Bio-Rad) and then blocked with 5% skim milk for 1 h. The membranes were then incubated with primary antibodies at 4°C for 12 h. After three washes in TBST, the membranes were incubated with horseradish peroxidase-conjugated secondary antibodies for 1 h at room temperature. After three additional washes with TBST, the membranes were detected with the SuperSignal West Pico chemiluminescence substrate (Pierce). The same blots were probed with a GAPDH antibody to normalize the protein level. Anti-PTPRG and anti-GAPDH antibodies were purchased from Santa Cruz Biotechnology (sc-1111; CA, USA) and Santa Cruz Biotechnology (B-7; CA, USA), respectively.

### Cell viability assay

MCF-7 and MDA-231 cells were seeded into 96-well plates at a density of at 5 × 10^4^ cells per well in 100 μL of culture medium, and then were incubated overnight in DMEM or L-15 medium supplemented with 10% FBS. The cells were collected 12, 24, 36, 48 and 60 h after transfection according to the manufacturer's instructions. After transfection, 10 μL of Cell Counting Kit-8 solution (CK04-500, Dojindo) was added into the corresponding test wells, and the cells were incubated for 3 h. Absorbance was measured at a wavelength of 450 nm. All experiments were performed in triplicate.

### Cell migration assay

Cell migration experiments were performed using 24-well matrigel-coated chambers (BD Biosciences, Bedford, MA, USA), containing an 8-μm-pore-sized membrane with a thin layer of Matrigel. MCF-7 and MDA- 231 cells were transfected and harvested as mentioned above, and were seeded at a density of 2 × 10^4^ per well on the upper chamber with serum-free DMEM or L-15. Complete medium was added into the lower compartment as a chemo-attractant. The cells were allowed to migrate for 8 hours. The remaining cells were scraped out from the upper face of the filters by cotton swabs. The Matrigel membranes were fixed with ice-cold methanol and stained with 0.1% crystal violet solution. Images of three different 20× fields of view were captured from each membrane. The number of invading cells was counted. All experiments were performed in triplicate and repeated twice.

### Apoptosis assays

Apoptosis of the MCF-7 and MDA-231 cells was tested by an Annexin V-FITC/propidium iodide (PI) staining assay. MCF-7 and MDA-231 cells were cultured in 12-well plates and transfected with pre-miR-19b, anti-miR-19b, PTPRG siRNA, or the PTPRG overexpression plasmid to induce apoptosis. The pre-miR-control, anti-miR-control, control siRNA, and a control plasmid served as the negative controls. The cells were harvested after being cultured for 24 h in DMEM or L-15 with 2% fetal bovine serum (Gibco). Flow cytometric analysis was performed using an Annexin V-FITC/PI staining kit (BD Biosciences, CA, USA) to detect cell apoptosis. After washing with cold PBS, the cells were resuspended in binding buffer (100 mM HEPES, pH 7.4, 100 mM NaCl, and 25 mM CaCl_2_), followed by staining with Annexin V-FITC/PI at room temperature in the dark for 15 min. Then, the apoptotic cells were assessed by gating the PI- and Annexin V-positive cells using a fluorescence-activated cell-sorting (FACS) flow cytometer (BD Biosciences, San Jose, CA, USA). All experiments were performed in triplicate.

### Statistical analysis

The results are shown as the means ± SEMs of at least three independent experiments. The quantitative RT-PCR, luciferase reporter assay and cell viability and apoptosis assays were performed in triplicate, and each individual experiment was repeated several times. All images are representative of at least three independent experiments. The differences were considered to be statistically significant at *P* < 0.05 using Student's *t*-test.

## SUPPLEMENTARY MATERIALS TABLE AND FIGURE


