# Supplementary material for: MiR-19b suppresses PTPRG to promote breast tumorigenesis
Source: Oncotarget. 2016 Sep 1;7(39):64100–8. doi: 10.18632/oncotarget.11799 (PMC5325428; doi:10.18632/oncotarget.11799)
Supplement: Supplementary file 1 [file oncotarget-07-64100-s001.pdf]

MiR-19b suppresses PTPRG to promote breast tumorigenesis

Supplementary Materials

Supplementary Table S1: Patients' characteristics

Case No.	Clinical History	Gender	TNM Stage
BCT #1	IDC	Female	II
BCT #2	IDC	Female	II
BCT #3	IDC	Female	II
BCT #4	IDC	Female	II
BCT #5	IDC	Female	II
BCT #6	IDC	Female	III
BCT #7	IDC	Female	II–III
BCT #8	IDC	Female	II–III
BCT #9	IDC	Female	I–II
BCT#10	IDC	Female	II
BCT#11	IDC	Female	II
BCT#12	IDC	Female	II
BCT#13	IDC	Female	II
BCT#14	IDC	female	II

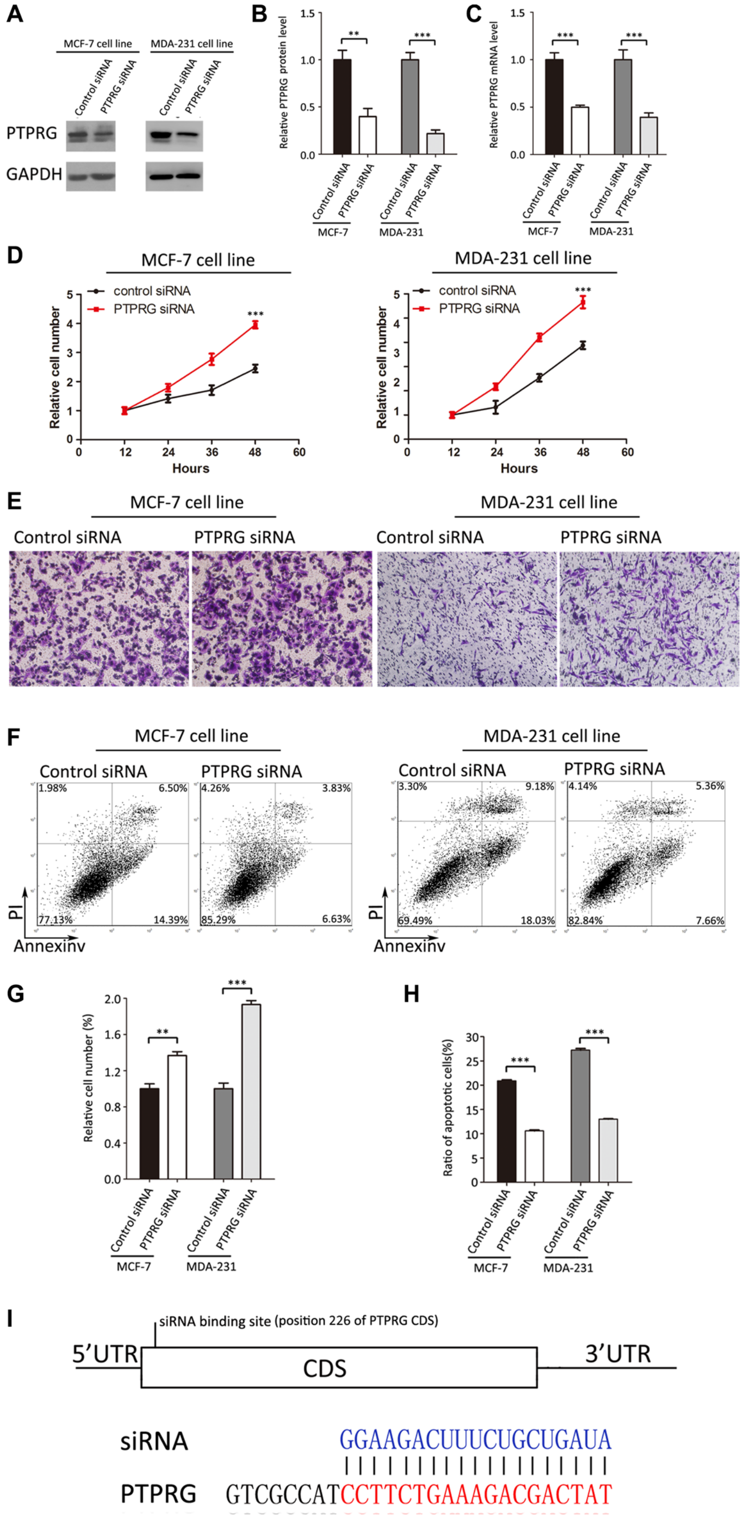

Supplementary Figure S1: Effects of PTPRG expression on the proliferation, migration and apoptosis of MCF-7 cells. (**A** and **B**) Western blotting analysis of the PTPRG protein levels in MCF-7 cells and MDA-231 cells treated with scrambled control siRNA, PTPRG siRNA. (**C**) Quantitative RT-PCR analysis of PTPRG mRNA levels in those cells treated with scrambled control siRNA, PTPRG siRNA. (**D**) CCK8 cell viability assays were performed 12, 24, 36 and 48 h after the transfection of the MCF-7 cells with either scrambled control siRNA or PTPRG siRNA. (**E** and **G**) Transwell migration assays were performed after the transfection of those cells with scrambled control siRNA or PTPRG siRNA. (**F** and **H**) Apoptosis assays were performed after the transfection of those cells with scrambled control siRNA or PTPRG siRNA. (**I**) Schematic diagram for knockdown of PTPRG by siRNs. ***P < 0.001; **P < 0.01.
